# Racial/ethnic Variations in the Association Between Financial Strain and Well-Being: Evidence from the United Kingdom Household Longitudinal Survey

**DOI:** 10.1007/s11482-025-10502-5

**Published:** 2025-10-24

**Authors:** Lei Chai, Cheng Chow, Zhuofei Lu

**Affiliations:** 1 Department of Social Work and Social Administration, The University of Hong Kong, Hong Kong, China; 2 School of Social Work, University of Texas at Austin, Austin, TX USA; 3 Department of Sociology, University of Oxford, Oxford, UK

**Keywords:** Financial strain, Mental health, Life satisfaction, Race/ethnicity

## Abstract

Financial strain is a major social stressor that adversely affects well-being, yet few studies have comprehensively examined whether its association differs by race/ethnicity. This study investigates whether the relationship between financial strain and two key outcomes—mental health and life satisfaction—varies across racial/ethnic groups using longitudinal data. Drawing on 13 waves of the United Kingdom Household Longitudinal Survey (UKHLS), spanning 2009–2010 to 2021–2022 and comprising 58,029 individuals and 417,766 person-years, we apply fixed-effects regression models to assess within-individual changes over time. Results show that financial strain is consistently associated with poorer mental health and lower life satisfaction. However, racial/ethnic variation emerges only in relation to mental health. Specifically, Black adults experience a stronger adverse association between financial strain and mental health compared to White adults. Subgroup analyses reveal further nuance: relative to White adults, African adults exhibit a more pronounced adverse association, whereas the association is not significant among Caribbean adults. Among Asian adults, financial strain is more strongly associated with poorer mental health for Indian individuals but less so for Chinese individuals. These findings highlight the need for tailored financial and mental health support initiatives that account for differences across both broad and specific racial/ethnic groups relative to the majority population. Targeted interventions may help mitigate the unequal burden of perceived financial stress and promote more equitable well-being outcomes.

**Supplementary Information:**

The online version contains supplementary material available at 10.1007/s11482-025-10502-5.

## Introduction

Financial strain refers to the perceived difficulty individuals face in meeting their financial needs (Bierman et al., 2023; Chai et al., 2021; Upenieks & Ellison, 2022). It encompasses challenges such as paying bills, affording healthcare, and covering household necessities, as well as a general sense of how well one’s finances hold up by the end of the month (Bierman et al., 2023; Chai et al., 2021; Upenieks & Ellison, 2022). This conceptualization highlights financial strain as a subjective experience rather than an objective measure of economic resources. Although financial strain is correlated with indicators such as income, assets, or poverty status, it remains conceptually distinct (Ettman et al., 2023). For instance, individuals with middle-class incomes may still experience financial strain when confronted with unanticipated expenses—such as medical bills or childcare costs—or the pressures of maintaining a particular lifestyle (Chai & Lu, 2025; Odle-Dusseau et al., 2018).

The subjective nature of financial strain is especially important, as studies consistently find that perceived financial stress can impact mental health outcomes as much as—or even more than—objective economic conditions (Camisasca et al., 2023; Ettman et al., 2023; Wilkinson, 2016). A well-documented case occurred during the Great Recession (2007–2009), when asset levels among older U.S. adults declined, yet reported financial strain and depression also decreased (Wilkinson, 2016). This paradox likely reflected social comparison processes: individuals evaluated their financial well-being relative to others who were similarly affected, reducing perceptions of strain and distress (Wilkinson, 2016). Such findings underscore the divergence between financial strain and objective hardship, affirming that perceived strain is a meaningful construct in its own right. Recent UK data further highlight the prevalence and relevance of financial strain. According to the Financial Conduct Authority (2024), approximately 7.4 million people in the UK report difficulties such as managing bill payments or repaying credit—underscoring the need to better understand the implications of financial strain for well-being.

Extensive research links financial strain to poorer mental health outcomes (Acevedo et al., 2014; Chai, 2023; Chai et al., 2021; Choi et al., 2023; Upenieks & Ellison, 2022). A smaller but growing body of work also associates financial strain with lower life satisfaction (Jung, 2018; Walsh, 2024). However, much of this literature is based on cross-sectional studies (e.g., Chai, 2023; Upenieks & Ellison, 2022; Walsh, 2024) or population-averaged models (e.g., generalized estimating equations in Choi et al., 2023) that do not account for unobserved, time-invariant characteristics such as personality. These factors may confound observed associations between financial strain and well-being, limiting the validity and precision of prior findings.

In addition to these methodological limitations, another key gap in the literature is the tendency to examine mental health and life satisfaction in isolation. While prior research has explored how financial strain relates to either outcome, few studies have considered both simultaneously. This is important because mental health and life satisfaction, though related, represent conceptually distinct dimensions of well-being. Mental health typically captures emotional and psychological functioning—such as symptoms of depression or anxiety—whereas life satisfaction reflects a broader and more stable cognitive evaluation of one’s overall life circumstances (Pavot & Diener, 2008). Prior research has variously framed them as separate dimensions of psychological functioning (Crosnoe et al., 2023), components of subjective well-being (Simon & Caputo, 2019), or related but independent constructs within mental health frameworks (Erving & Thomas, 2018). By analyzing both outcomes together, this study offers a more comprehensive understanding of how financial strain shapes well-being and allows for the detection of potentially divergent effects that would be missed by examining either outcome in isolation.

Understanding these relationships also requires consideration of social context. The stress process model (Pearlin & Bierman, 2013) emphasizes that exposure to stressors and access to coping resources are socially patterned and unequally distributed across groups. Racial and ethnic minorities are more likely to experience chronic stressors like financial strain and less likely to have access to protective resources such as strong social support networks (Lee & Turney, 2012). The minority stress framework (Meyer, 2003; Nie, 2024) further emphasizes how exposure to minority-specific stressors (e.g., discrimination, internalized stigma) may compound the psychological toll of financial strain. Despite these theoretical insights, few studies have empirically tested whether the association between financial strain and well-being varies by racial/ethnic group—especially using longitudinal data. This remains a critical gap, as understanding whether racial/ethnic disparities shape the effects of financial strain over time can help identify population-specific vulnerabilities and guide the development of more equitable policy interventions.

To address these gaps, the present study uses 13 waves (2009–2022) of the UK Household Longitudinal Study (UKHLS) to examine how financial strain relates to both mental health and life satisfaction, and whether these associations vary across racial/ethnic groups. We employ fixed-effects regression models to estimate within-person changes, minimizing bias from time-invariant individual characteristics such as personality. While this approach cannot eliminate all sources of endogeneity (e.g., time-varying confounders or reverse causality), it offers a more rigorous assessment of the relationship between changes in financial strain and changes in well-being—and of the moderating role of race/ethnicity—than cross-sectional or population-averaged methods.

## Background

### Financial Strain and Mental Health

Mental health, given its sensitivity to social and economic conditions, is particularly vulnerable to the effects of financial strain—often more so than physical health (Ettman et al., 2023). While improving objective economic conditions is essential for reducing population-level mental health burdens, a growing body of research highlights the importance of understanding financial strain as a subjective, chronic stressor in its own right (Ettman et al., 2023). The stress process model provides a useful theoretical lens for this relationship (Pearlin & Bierman, 2013), emphasizing how both acute and chronic stressors can lead to psychological distress depending in part on access to coping resources. Within this framework, financial strain is understood as a chronic stressor that gradually erodes psychological and social resources (Bierman et al., 2023; Chai & Lu, 2025; Upenieks & Ellison, 2022). Over time, the depletion of these psychosocial resources—including a sense of control, self-worth, and social support—can increase vulnerability to emotional distress and mental health decline (Pearlin & Bierman, 2013). For instance, individuals experiencing financial strain often report persistent worry, frustration, and a loss of control—emotions closely tied to poor mental health outcomes (Choi et al., 2021). The uncertainty associated with financial instability can also diminish self-worth and foster feelings of helplessness (Acevedo et al., 2014; Hughes et al., 2014). Moreover, financial strain may hinder the formation or maintenance of social connections due to shame or lack of social support resources, potentially leading to social isolation and worsened mental health (Acevedo et al., 2014).

Although many studies have documented a link between financial strain and mental health in general adult populations, much of this research relies on cross-sectional data (Acevedo et al., 2014; Upenieks & Ellison, 2022). Some studies have expanded the focus to specific subgroups, including adolescents (Park & Lee, 2023; Yang et al., 2023), young adults (Chen et al., 2024), and older adults (Bierman et al., 2023; Chai, 2023; Kong et al., 2024). While recent longitudinal studies offer valuable insights, methodological limitations persist. For instance, Choi and colleagues (2023) used the Korean Welfare Panel Study to demonstrate a link between financial strain and depression, but their use of generalized estimating equations (GEE) captured only population-averaged effects and did not control for unobserved, time-invariant individual characteristics—limiting their ability to assess within-individual changes. Similarly, Hertz-Palmor and colleagues (2021) conducted a two-wave U.S. study and found a link between financial strain and depressive symptoms over one month. However, their snowball sampling method—recruiting participants online through social networks—likely underrepresented low-income individuals with limited internet access, potentially biasing results away from the most disadvantaged populations (Hertz-Palmor et al., 2021). These limitations raise questions about the robustness of prior findings and restrict our understanding of how changes in financial strain over time shape individuals’ mental health outcomes.

### Financial Strain and Life Satisfaction

In addition to mental health, financial strain also appears to shape life satisfaction—a core component of subjective well-being (Jung, 2018; Walsh, 2024). Life satisfaction reflects individuals’ overall evaluation of their lives, encompassing multiple domains such as health, relationships, and economic security (Kim & Park, 2024). According to need theory, life satisfaction depends largely on the ability to meet basic needs such as having adequate food, housing, and clothing (Diener & Biswas-Diener, 2002; Jung, 2018). When individuals perceive themselves as unable to meet these needs, they tend to report lower satisfaction with life. Financial strain can also obstruct the pursuit of personal goals, which is particularly detrimental given the strong link between goal attainment and life satisfaction (Emmons, 1986). Persistent financial challenges may further erode self-concept, especially when individuals compare themselves unfavorably to others in terms of material well-being (Bradshaw & Ellison, 2010). These unfavorable social comparisons can diminish self-esteem and contribute to reduced life satisfaction (Zagefka & Brown, 2005).

Life satisfaction has also been framed within the stress process model (Kim & Park, 2024), which views it as a broad cognitive and emotional appraisal of well-being (Jung, 2018). From this perspective, financial strain represents a key stressor that can negatively influence both mental health and life satisfaction. While cross-sectional studies using data from the European Social Survey and the World Values Survey have documented a significant association between financial strain and lower life satisfaction (Jung, 2018; Walsh, 2024), few studies have explored this relationship using longitudinal data—a gap this study seeks to address.

Taken together, existing research points to two key limitations: the tendency to examine mental health and life satisfaction in isolation, and the widespread use of analytical approaches that cannot account for unobserved heterogeneity. Addressing these gaps is not just a methodological concern—it is essential for clarifying how financial strain shapes well-being across multiple dimensions and for identifying which populations may be most affected. Mental health and life satisfaction, while correlated, reflect distinct aspects of well-being—emotional functioning versus evaluative judgments of quality of life. Analyzing them together enables a more comprehensive assessment of whether financial strain exerts consistent or divergent effects. At the same time, understanding how these associations unfold within individuals over time requires methods that move beyond population averages. By applying fixed-effects regression models, this study isolates within-person changes in financial strain and well-being, offering stronger evidence than prior cross-sectional or population-averaged studies.

### Racial/Ethnic Variations

While growing attention has been paid to the relationship between financial strain and both mental health and life satisfaction, relatively little is known about how these associations vary across racial/ethnic groups. Yet, both the stress process model (Pearlin & Bierman, 2013) and the minority stress framework (Meyer, 2003) suggest that such variation is theoretically meaningful and may illuminate important dimensions of inequality. Understanding whether and how financial strain operates differently across racialized populations is therefore essential to uncovering population-specific vulnerabilities and informing equitable policy responses.

The stress process model emphasizes that exposure to stressors—and access to coping resources—is not evenly distributed (Pearlin & Bierman, 2013). Individuals from marginalized groups, including racial/ethnic minorities, are often disproportionately burdened by chronic stressors such as financial strain and simultaneously have fewer resources, such as social support, to buffer these effects (Lee & Turney, 2012). This unequal distribution reinforces disparities in well-being, as groups with less power and privilege experience greater psychological tolls and fewer opportunities for recovery. Building on this, the minority stress framework highlights the additional stress burden experienced by individuals who occupy socially marginalized positions (Meyer, 2003). While originally developed to explain the mental health consequences of stigma among sexual minorities, the framework has since been extended to racial/ethnic disparities (De Leon et al., 2024; Nie, 2024). From this perspective, minorities are exposed to both general stressors (e.g., financial strain) and minority-specific stressors (e.g., racial discrimination, internalized stigma) (Meyer, 2003; Nie, 2024), which together compound psychological strain. For instance, certain Asian populations—despite relatively higher average socioeconomic status (Zhou & Lee, 2017)—often report elevated psychological distress due to persistent racism and stereotyping (Nie, 2024). This cumulative stress exposure can erode resilience and reduce coping capacity when faced with additional challenges like financial strain.

In the UK context, this theoretical perspective is particularly relevant given the country’s racial/ethnic diversity. According to the 2021 Census in England and Wales (Government of the UK, 2024), the population was 59.6 million, with 81.7% identifying as White. Asian communities represented the second-largest group (9.3%), followed by Black (4%), Mixed (2.9%), and Other racial/ethnic groups (2.1%). Within this context, racial/ethnic minority groups experience stressors unevenly. For instance, Black African and Caribbean populations face high levels of socioeconomic-related stressors, including persistent poverty, unemployment, and insecure housing (Department for Work & Pensions [DWP], 2023; Jeffery et al., 2024). These groups, along with some South Asian communities—particularly Bangladeshi and Pakistani individuals—also report higher exposure to race-based stressors, such as racial discrimination and unequal treatment in housing, employment, and public services (Wallace et al., 2016). Conversely, while the Indian community is generally better off socioeconomically, individuals may continue to experience stress related to cultural obligations, such as the expectation to support extended family networks (Thandi, 2019). These culturally specific demands may not always register in standard economic indicators but can significantly shape perceived strain.

In addition to disparities in stress exposure, unequal access to coping resources may further influence the effects of financial strain (Pearlin & Bierman, 2013). Socioeconomic disadvantage, in particular, can constrain individuals’ ability to access and mobilize psychosocial resources. For instance, individuals employed in precarious, low-wage jobs—often with irregular schedules or limited autonomy—may have fewer opportunities to engage in stress-relieving activities or draw on community networks (Utzet et al., 2020). This pattern is particularly common among Pakistani and Bangladeshi adults in the UK (Hackett et al., 2020; McDowell et al., 2014). Beyond material hardship, cultural norms and family structures also shape coping capacity. In some East and South Asian communities, strong intergenerational obligations can provide a source of identity and support but may also heighten stress when financial strain limits individuals’ ability to meet these expectations (Batnitzky et al., 2012). While such values may enhance resilience for some, they can become an added burden in times of economic instability.

Social support networks also vary in structure and accessibility across racial/ethnic groups, shaping the availability of coping resources. For instance, African and Caribbean communities often rely on extended family and faith-based organizations, which can serve as emotional buffers; however, these networks may become strained under economic hardship, limiting their effectiveness as sources of support (Burholt et al., 2018). Moreover, stigma surrounding mental health within some of these communities may discourage help-seeking and reduce access to formal coping support (Hamid & Furnham, 2013; Ho et al., 2021). Collectively, these differences help explain why financial strain may have divergent psychological effects across racial/ethnic groups.

Despite these theoretical insights, empirical research on racial/ethnic disparities in the effects of financial strain remains limited. Studies have shown that racial/ethnic minorities, particularly Black Americans, experience intensified mental health effects of financial strain due to structural racism and persistent discrimination (Hughes et al., 2014). For instance, cross-sectional data from the 2001–2003 National Survey of American Life documented a strong association between financial strain and depressive symptoms among Black adults (Hughes et al., 2014). However, these findings are now dated, and most relevant studies remain U.S.-focused. This represents a notable limitation, as the UK differs from the U.S. in racial/ethnic composition, welfare state structure, healthcare access, and anti-discrimination policies (Exworthy et al., 2006; Fieldhouse & Cutts, 2010; Li & Heath, 2016; McAreavey & Brown, 2019). These institutional and demographic differences may shape both the experiences and consequences of financial strain. Yet, relatively few UK-based studies have explored how financial strain interacts with racial/ethnic disparities in well-being.

Moreover, while mental health has received more empirical attention, life satisfaction remains underexamined in the context of racial/ethnic disparities in financial strain. As interest grows in understanding the full scope of well-being inequality, it becomes increasingly important to assess both mental health and life satisfaction across racial/ethnic groups. Most existing studies rely on cross-sectional designs (e.g., Hughes et al., 2014), limiting our ability to assess how these associations evolve over time. Longitudinal research is therefore needed to capture the dynamic nature of financial strain’s effects and uncover population-specific vulnerabilities.

### The Present Study

Building on the limitations and gaps identified above, the present study uses 13 waves (2009–2022) of the UK Household Longitudinal Study (UKHLS) to examine how changes in financial strain relate to two key well-being outcomes—mental health and life satisfaction—and whether these associations differ across racial/ethnic groups. We apply fixed-effects regression models to assess within-individual changes over time, thereby minimizing bias from unobserved, time-invariant characteristics. We test two hypotheses: Financial strain is associated with poorer mental health and lower life satisfaction (Hypothesis 1). The adverse association between financial strain and both mental health and life satisfaction is stronger among racial/ethnic minorities compared to White individuals (Hypothesis 2).

## Methods

### Data

This research utilizes the UK Household Longitudinal Study (UKHLS), a large-scale, nationally representative survey conducted in the United Kingdom. Initially launched in 2009, the survey collected data from a clustered and stratified sample of approximately 50,000 individuals across 30,000 households, all interviewed in person. Additional samples have been added in subsequent waves to maintain the dataset’s representativeness. These Households have been re-surveyed annually, resulting in 13 waves from 2009 to 2010 to 2021–2022 (Institute for Social and Economic Research, 2023). All participants provided informed consent, and the data were fully anonymized before public release. As this study draws on de-identified, publicly accessible data, no ethical review was required. The analytical sample is restricted to individuals aged 18 and older.

We address two types of missing data: item nonresponse and attrition. First, item-level missingness was minimal for most variables, ranging from approximately 0.07% (total net monthly personal income) to 5.73% (financial strain). However, missingness for the two outcome variables—mental health (GHQ-12) and life satisfaction—was approximately 13% of person-year observations, exceeding the commonly cited 10% threshold. Although multiple imputation (MI) is often recommended in such cases, we opted not to use it because one of the key assumptions underlying MI—the missing-at-random (MAR) mechanism—may not hold in our data. In particular, missing values for mental health and life satisfaction were disproportionately concentrated in earlier waves, suggesting that the missingness may not be random. Given this, we used listwise deletion and excluded all person-wave observations with missing values for any analysis variable. Second, individuals who contributed data in only a single wave were excluded, as fixed-effects models require at least two observations per person to estimate within-individual change. After applying these exclusions, the final analytical dataset consists of 58,029 individuals and 417,766 person-years. The resulting panel is unbalanced, meaning individuals contributed to a varying number of observations depending on their participation across waves. This approach allows us to maximize sample size and statistical power by including all available within-person data without requiring complete participation across 13 waves.

### Measures

Mental health was assessed using the 12-item General Health Questionnaire (GHQ-12), which captures various aspects of psychological well-being, including concentration, sleep quality, sense of purpose, decision-making ability, stress levels, coping difficulty, enjoyment of daily activities, resilience, depressive symptoms, self-confidence, self-worth, and general happiness. Each item was originally coded on a 4-point Likert scale: better than usual (1), same as usual (2), less than usual (3), or much less than usual (4). Following prior research (Chai & Lu, 2025; Lu et al., 2023; Wang et al., 2022), items were recoded from 0 to 3. The total GHQ score was computed by summing the recoded items, resulting in a range from 0 to 36, with higher scores indicating poorer mental health.

Life satisfaction was measured using a single-item question: “How dissatisfied or satisfied are you with your life overall?” Responses were coded on a 7-point scale ranging from completely dissatisfied (1), mostly dissatisfied (2), somewhat dissatisfied (3), neither satisfied nor dissatisfied (4), somewhat satisfied (5), mostly satisfied (6), to completely satisfied (7). To maintain consistency with the GHQ scoring—where higher scores reflect worse outcomes—responses were reverse-coded, so that higher scores indicate lower life satisfaction. This single-item measure aligns with common practice in the literature (Jung, 2018; Walsh, 2024; Wang et al., 2022) and has been shown to yield valid and reliable results comparable to those from multi-item scales (Kim & Park, 2024).

Financial strain was assessed with the question (Chai & Lu, 2025; Chai et al., 2025; Wang et al., 2022): “How well would you say you are managing financially these days?” Responses ranged from living comfortably (1), doing alright (2), just about getting by (3), finding it quite difficult (4), to finding it very difficult (5), with higher scores indicating greater financial strain.

Race/ethnicity was recoded into the following categories (Maletta et al., 2023, 2025): White (British, English, Scottish, Welsh, Northern Irish, Irish, or any other White background), Mixed (White and Black Caribbean, White and Black African, White and Asian, or any other mixed background), Asian (Indian, Pakistani, Bangladeshi, Chinese, or any other Asian background), Black (Caribbean, African, or any other Black background), and Other (Arab or any other racial/ethnic group).

We included several time-varying sociodemographic controls, given their potential influence on both financial strain and well-being (Bierman et al., 2023; Chai & Lu, 2025; Chai et al., 2025; Cho et al., 2021; Jung, 2018). To avoid adjusting for potential mediators, we limited controls to core sociodemographic factors. Marital status was dichotomized as married or not married. Number of children under age 15 was treated as a continuous variable. Educational attainment was categorized as: degree (equivalent to a university bachelor’s degree), other higher degree (e.g., postgraduate diplomas or master’s degrees), A-level (advanced qualifications typically obtained at age 18 and required for university entry), GCSE (General Certificate of Secondary Education, usually completed at age 16), other qualification (e.g., vocational certificates or foreign credentials), and no qualification (no formal educational credentials). Employment status was recoded as employed or other (e.g., unemployed, retired, or full-time student). Total net monthly personal income was treated as a continuous variable. Survey wave was coded from 1 to 13.

### Analytical Strategy

This study employs fixed-effects regression models to examine how within-individual changes in financial strain correspond to changes in mental health and life satisfaction over time, with a focus on potential racial/ethnic differences. By controlling for unobserved, time-invariant individual characteristics, fixed-effects models mitigate concerns about confounding and allow for stronger causal inference than cross-sectional approaches.

The general fixed-effects equation is specified as follows:

$$\:{Y}_{it}={\alpha\:}_{t}+\beta\:1{Financial\:strain}_{it}+\beta\:2{Covariates}_{it}+{T}_{t}+{\mu\:}_{i}+{\epsilon\:}_{it}$$

where $\:{Y}_{it}$ represents the dependent variable (mental health or life satisfaction) for individual i at time t (t = 1, 2, …, *T*). The term $\:{\alpha\:}_{t}$ denotes the time-specific intercept, and $\:\beta\:1$ captures the effect of financial strain. The vector $\:\beta\:2$ contains coefficients for the time-varying covariates. $\:{T}_{t}$ represents time effects, and $\:{\mu\:}_{i}$ captures unobserved, time-invariant individual heterogeneity (eliminated in fixed-effects estimation), and $\:{\epsilon\:}_{it}$ is the time-varying error term.

The regression models are structured as follows: Table 1 presents fixed-effects models estimating the association between financial strain and mental health (Model 1) and life satisfaction (Model 2) for the full sample. These models control for time-varying covariates, including marital status, number of children, education, employment status, personal income, and survey year. Table 2 extends this analysis by stratifying the sample into racial/ethnic groups: White (Model 1), Mixed (Model 2), Asian (Model 3), Black (Model 4), and Other (Model 5). Model 6 includes interaction terms to formally test whether the associations between financial strain and well-being differ significantly across these groups. Table 3 further disaggregates the analysis by examining eleven more specific racial/ethnic subgroups—White, White and Caribbean, White and African, White and Asian, Indian, Pakistani, Bangladeshi, Chinese, Caribbean, African, and Arab. Models 1–11 estimate associations separately for each subgroup, while Model 12 includes interaction terms to test whether these associations differ significantly from those observed among White individuals. Tables 2 and 3 therefore provide complementary perspectives. The aggregated categories in Table 2 capture broad racial/ethnic disparities, while the disaggregated subgroups in Table 3 uncover variation that may be masked in aggregated analyses. Taken together, this two-level approach avoids overgeneralization and provides a fuller understanding of both overall patterns and subgroup-specific differences, with White serving as the reference group in both cases.

**Table 1:** Fixed-effects regression models predicting mental health and life satisfaction

	Model 1 Mental health b	Model 2 Life satisfaction b
Financial strain	1.027***	0.251***
	(0.010)	(0.003)
Individuals	58,029	58,029
Person-years	417,766	417,766

All models include a full set of control variables: marital status, number of children, education, employment status, personal income (logged), and survey-year. Education was recoded into a binary variable, distinguishing individuals with a bachelor’s degree or higher from those with lower educational attainment, to ensure sufficient cell sizes when examining racial/ethnic differences. b = coefficient

Standard errors are in parentheses. ***p <.001

The interaction terms are used to test for effect modification by race/ethnicity—that is, whether the relationship between financial strain and well-being outcomes differ significantly across groups. This is a standard and widely accepted approach in social science research to assess heterogeneity in associations. Although interaction effects are sometimes visualized using graphs, in this case the number of groups (five in Table 2 and eleven in Table 3) would produce overly crowded and difficult-to-interpret figures. For clarity and readability, we therefore present interaction effects in regression tables, which remain a common and appropriate method of presentation.

**Table 2:** Fixed-effects regression models predicting mental health and life satisfaction

	Model 1	Model 2	Model 3	Model 4	Model 5	Model 6
	Top panel: Mental health					
	White b	Mixed b	Asian b	Black b	Other b	Full b
Financial strain	1.016***	1.123***	1.058***	1.096***	1.133***	1.014***
	(0.011)	(0.082)	(0.036)	(0.053)	(0.133)	(0.011)
Financial strain X race/ethnicity						
Financial strain X Mixed						0.080
						(0.072)
Financial strain X Asian						0.056
						(0.034)
Financial strain X Black						0.108*
						(0.049)
Financial strain X Other						0.167
						(0.117)

	Bottom panel: Life satisfaction					
	White b	Mixed b	Asian b	Black b	Other b	Full b
Financial strain	0.251***	0.278***	0.238***	0.265***	0.214***	0.250***
	(0.003)	(0.021)	(0.010)	(0.015)	(0.036)	(0.003)
Financial strain X race/ethnicity						
Financial strain X Mixed						0.025
						(0.021)
Financial strain X Asian						− 0.005
						(0.010)
Financial strain X Black						0.025
						(0.014)
Financial strain X Other						− 0.020
						(0.033)
Individuals	48,076	1,084	5,864	2,548	457	58,029
Person-years	361,161	6,737	33,339	14,074	2,455	417,766

All models include a full set of control variables: marital status, number of children, education, employment status, personal income (logged), and survey-year. Education was recoded into a binary variable, distinguishing individuals with a bachelor’s degree or higher from those with lower educational attainment, to ensure sufficient cell sizes when examining racial/ethnic differences. b = coefficient

Standard errors are in parentheses. ***p <.001; *p <.05

To determine whether fixed-effects models were more appropriate than random-effects models, Hausman tests were conducted (Allison, 2009). Results from these tests indicated that random-effects models would likely have introduced bias due to correlations between predictors and unobserved, time-invariant individual characteristics. Therefore, fixed-effects models were selected as the preferred analytical approach. Two exceptions emerged: the Hausman test for Model 4 (White and Asian) and Model 8 (Chinese) of Table 3 in the top panel (mental health) suggested that a random-effects model may be preferred. However, because the coefficients were substantively similar across both models and fixed-effects were favored in most cases, we retained fixed-effect estimates throughout for consistency. All statistical analyses were conducted using Stata 19.

**Table 3:** Fixed-effects regression models predicting mental health and life satisfaction

	Model 1	Model 2	Model 3	Model 4	Model 5	Model 6	Model 7	Model 8	Model 9	Model 10	Model 11	Model 12
	Top panel: Mental health											
	White	White & Caribbean b	White & African b	White & Asian b	Indian b	Pakistani b	Bangladeshi b	Chinese b	Caribbean b	African b	Arab b	Full b
Financial strain	1.016***	1.351***	1.141***	0.752***	1.108***	1.120***	1.087***	0.597***	1.065***	1.129***	1.342***	1.014***
	(0.011)	(0.132)	(0.213)	(0.162)	(0.058)	(0.067)	(0.090)	(0.169)	(0.079)	(0.073)	(0.189)	(0.011)
Financial strain X race/ethnicity												
Financial strain X White & Caribbean												0.321**
												(0.112)
Financial strain X White & African												0.097
												(0.197)
Financial strain X White & Asian												− 0.314*
												(0.148)
Financial strain X Indian												0.110*
												(0.054)
Financial strain X Pakistani												0.105+
												(0.056)
Financial strain X Bangladeshi												0.100
												(0.077)
Financial strain X Chinese												− 0.433*
												(0.175)
Financial strain X Caribbean												0.096
												(0.073)
Financial strain X African												0.140*
												(0.068)
Financial strain X Arab												0.382*
												(0.167)

	Bottom panel: Life satisfaction											
	White	White & Caribbean b	White & African b	White & Asian b	Indian b	Pakistani b	Bangladeshi b	Chinese b	Caribbean b	African b	Arab b	Full b
Financial strain	0.251***	0.278***	0.232***	0.276***	0.254***	0.219***	0.264***	0.278***	0.260***	0.262***	0.200***	0.250***
	(0.003)	(0.033)	(0.061)	(0.041)	(0.016)	(0.018)	(0.025)	(0.046)	(0.023)	(0.021)	(0.054)	(0.003)
Financial strain X race/ethnicity												
Financial strain X White & Caribbean												0.030
												(0.032)
Financial strain X White & African												− 0.007
												(0.056)
Financial strain X White & Asian												0.016
												(0.042)
Financial strain X Indian												0.013
												(0.015)
Financial strain X Pakistani												− 0.023
												(0.016)
Financial strain X Bangladeshi												0.018
												(0.022)
Financial strain X Chinese												0.022
												(0.050)
Financial strain X Caribbean												0.026
												(0.021)
Financial strain X African												0.021
												(0.019)
Financial strain X Arab												− 0.049
												(0.047)
Individuals	48,076	430	159	255	2,155	1,832	1,024	238	1,102	1,354	235	58,029
Person-years	361,161	2,694	911	1,685	13,008	9,851	5,211	1,643	6,535	6,981	1,193	417,766

All models include a full set of control variables: marital status, number of children, education, employment status, personal income (logged), and survey-year

Education was recoded into a binary variable, distinguishing individuals with a bachelor’s degree or higher from those with lower educational attainment, to ensure sufficient cell sizes when examining racial/ethnic differences. b = coefficient. Standard errors are in parentheses. ***p <.001; **p <.01; *p <.05; +*p* <.10.

The following categories were excluded due to limited table space and challenges in interpreting these categories: any other Mixed, any other Asian, any other Black, and any other race/ethnicity. Due to smaller subgroup sizes in Table 5, a significance threshold of 0.10 was used, as smaller samples reduce statistical power to detect effects at the conventional 0.05 level

## Results

Descriptive statistics for the full sample and each racial/ethnic group (White, Mixed, Asian, Black, and Other) are presented in Table 4. Additional descriptive statistics for more detailed racial/ethnic subgroups are provided in Table 5. These tables are based on the full set of person-year observations included in the analysis (i.e., pooled person-wave data). This approach aligns with our use of fixed-effects regression models and ensures that the descriptive tables reflect the structure of the analytical sample.

Table 4 presents descriptive statistics for the full sample and broad racial/ethnic groups. Significant racial/ethnic differences were observed across all three key variables: mental health, life satisfaction, and financial strain. Post-hoc Tukey tests indicated that compared to White respondents (mean = 11.20), Mixed (mean = 12.15), Asian (mean = 11.48), and Other (mean = 12.14) groups reported significantly poorer mental health (i.e., higher GHQ-12 scores), while Black respondents (mean = 10.87) reported significantly better mental health. Life satisfaction scores—where higher values indicate lower satisfaction—were significantly higher among all racial/ethnic minority groups compared to White respondents (mean = 2.79), suggesting lower evaluative well-being across these groups. Financial strain was also significantly more pronounced among racial/ethnic minorities, with Black (mean = 2.74), Asian (mean = 2.39), Mixed (mean = 2.44), and Other (mean = 2.49) respondents all reporting higher average levels of strain than White respondents (mean = 2.05).

**Table 4:** Descriptive statistics of selected variables in the analysis

	Full sample		White		Mixed		Asian		Black		Other		Significance	
	Mean/%	SD	Mean/%	SD	Mean/%	SD	Mean/%	SD	Mean/%	SD	Mean/%	SD		
Mental health	11.23	5.55	11.20	5.47	12.15	6.33	11.48	5.95	10.87	5.89	12.14	6.05	***	
Life satisfaction	2.83	1.46	2.79	1.44	3.19	1.56	3.04	1.52	3.10	1.56	3.11	1.51	***	
Financial strain	2.11	0.99	2.05	0.97	2.44	1.06	2.39	1.05	2.74	1.05	2.49	1.11	***	
Marital status														
Married	55.27		55.33		34.78		66.50		36.86		56.09		***	
Unmarried	44.73		44.67		65.22		33.50		63.14		43.91			
Number of children	0.27	0.71	0.25	0.67	0.35	0.76	0.45	0.92	0.45	0.91	0.44	0.89	***	
Education													***	
Degree	27.12		25.68		36.46		37.00		32.84		46.80			
Other higher	12.65		12.75		14.10		9.62		16.86		11.41			
A-level	20.93		20.88		25.49		21.07		20.71		15.11			
GCSE	19.34		20.04		15.69		15.14		14.64		10.47			
Other	9.15		9.46		5.09		6.93		8.45		8.72			
No qualification	10.81		11.19		3.18		10.22		6.50		7.49			
Employment status													***	
Employed	57.32		56.96		63.96		58.58		61.07		53.65			
Other	42.68		43.04		36.04		41.42		38.93		46.35			
Personal income	1566.77	2306.17	1591.27	2371.61	1545.32	1816.30	1349.14	1966.95	1475.95	1462.67	1498.72	1719.91	***	
Individuals	58,029		48,076		1,084		5,864		2,548		457			
Person-years	417,766		361,161		6,737		33,339		14,074		2,455			

To test for differences across racial/ethnic groups, we conducted ANOVA tests for continuous variables and chi-squared tests for categorical variables

Post-hoc Tukey tests showed that compared to White individuals, Mixed (*p* <.001), Asian (*p* <.001), and Other (*p* <.001) groups reported significantly higher mean mental health scores, indicating poorer mental health, while Black individuals (*p* <.001) reported significantly lower mean scores, indicating better mental health

For life satisfaction, Mixed (*p* <.001), Asian (*p* <.001), Black (*p* <.001), and Other (*p* <.001) all reported significantly higher mean scores, indicating lower life satisfaction, compared to White individuals. For financial strain, Mixed (*p* <.001), Asian (*p* <.001), Black (*p* <.001), and Other (*p* <.001) all reported significantly higher mean scores, indicating greater financial strain, compared to White individuals

Table 5 disaggregates these comparisons further by detailed racial/ethnic subgroups. Post-hoc Tukey tests indicated that several groups reported significantly poorer mental health compared to White respondents (mean = 11.20), including White and Caribbean (mean = 12.47), White and Asian (mean = 11.76), White and any other mixed (mean = 12.30), Pakistani (mean = 11.96), Bangladeshi (mean = 11.87), Caribbean (mean = 11.44), and Arab (mean = 12.33), any other group (mean = 11.96). In contrast, African respondents reported significantly better mental health (mean = 10.32), while Indian, Chinese, White and African, any other Asian, and any other Black respondents did not differ significantly from White respondents. For life satisfaction, all non-White groups reported significantly higher mean scores, indicating lower evaluative well-being compared to White respondents (mean = 2.79). For financial strain, nearly all non-White groups reported significantly higher mean scores, with the exception of Chinese respondents (mean = 2.00), who did not differ significantly from White respondents (mean = 2.05). These descriptive findings reinforce the importance of analyzing racial/ethnic variations in both emotional and evaluative aspects of well-being.

**Table 5:** Descriptive statistics of selected variables in the analysis

	White		White & Caribbean		White & African		White & Asian		White & any other mixed		Significance
	Mean/%	SD	Mean/%	SD	Mean/%	SD	Mean/%	SD	Mean/%	SD	
Mental health	11.20	5.47	12.47	6.63	11.75	6.01	11.76	6.11	12.30	6.19	***
Life satisfaction	2.79	1.44	3.30	1.64	3.30	1.53	2.95	1.44	3.17	1.54	***
Financial strain	2.05	0.97	2.58	1.05	2.53	1.07	2.20	1.05	2.39	1.02	***
Marital status											***
Married	55.33		24.09		35.35		44.63		42.85		
Unmarried	44.67		75.91		64.65		55.37		57.15		
Number of children	0.25	0.67	0.37	0.79	0.43	0.84	0.33	0.74	0.29	0.68	***
Education											***
Degree	25.68		28.43		35.78		43.26		43.88		
Other higher	12.75		13.21		14.71		15.01		14.31		
A-level	20.88		25.80		27.33		24.87		24.46		
GCSE	20.04		21.12		18.00		10.62		10.02		
Other	9.46		6.31		3.73		3.56		5.46		
No qualification	11.19		5.12		0.44		2.67		1.87		
Employment status											
Employed	56.96		59.21		68.28		66.29		67.38		***
Other	43.04		40.79		31.72		33.71		32.62		
Personal income	1591.27	2371.61	1435.10	1455.80	1602.26	1467.08	1581.59	1577.26	1672.46	2669.54	***
Individuals	48,076		430		159		255		240		
Person-years	361,161		2,694		911		1,685		1,447		

	Indian		Pakistani		Bangladeshi		Chinese		Any other Asian		
	Mean/%	SD	Mean/%	SD	Mean/%	SD	Mean/%	SD	Mean/%	SD	
Mental health	11.16	5.79	11.96	6.35	11.87	6.06	11.16	4.79	10.90	5.53	
Life satisfaction	2.96	1.49	3.12	1.58	3.15	1.53	2.98	1.28	2.95	1.47	
Financial strain	2.23	1.00	2.53	1.05	2.63	1.07	2.00	0.90	2.41	1.08	
Marital status											
Married	69.70		65.43		62.62		60.62		66.19		
Unmarried	30.30		34.57		37.38		39.38		33.81		
Number of children	0.35	0.77	0.58	1.10	0.52	1.00	0.28	0.64	0.40	0.80	
Education											
Degree	41.82		28.55		27.37		69.26		41.95		
Other higher	10.81		8.72		6.49		7.91		13.10		
A-level	19.62		22.76		25.87		10.10		19.80		
GCSE	13.06		19.24		18.77		4.93		10.89		
Other	6.40		7.68		6.64		2.92		9.07		
No qualification	8.29		13.05		14.87		4.87		5.18		
Employment status											
Employed	64.47		49.59		52.33		72.73		64.48		
Other	35.53		50.41		47.67		27.27		35.52		
Personal income	1521.22	2147.98	1083.30	1142.88	1170.31	2678.05	1919.24	1793.32	1452.76	1791.66	
Individuals	2,155		1,832		1,024		238		615		
Person-years	13,008		9,851		5,211		1,643		3,626		

	Caribbean		African		Any other Black		Arab		Any other race/ethnicity		
	Mean/%	SD	Mean/%	SD	Mean/%	SD	Mean/%	SD	Mean/%	SD	
Mental health	11.44	6.01	10.32	5.74	11.26	5.87	12.33	6.22	11.96	5.89	
Life satisfaction	3.22	1.60	2.99	1.52	3.10	1.41	3.14	1.49	3.08	1.52	
Financial strain	2.68	1.03	2.82	1.07	2.52	1.03	2.54	1.07	2.44	1.14	
Marital status											
Married	29.58		44.26		29.57		53.73		58.32		
Unmarried	70.42		55.74		70.43		46.27		41.68		
Number of children	0.32	0.72	0.58	1.05	0.41	0.78	0.48	0.98	0.40	0.79	
Education											
Degree	25.05		40.34		30.29		50.29		43.50		
Other higher	16.62		16.93		18.82		8.47		14.18		
A-level	19.50		21.11		29.93		18.69		11.73		
GCSE	19.13		10.86		9.32		9.39		11.49		
Other	10.73		6.42		7.17		5.95		11.33		
No qualification	8.98		4.34		4.48		7.21		7.77		
Employment status											
Employed	59.45		62.20		65.95		51.89		55.31		
Other	40.55		37.80		34.05		48.11		44.69		
Personal income	1508.45	1753.82	1443.67	1157.69	1499.29	1072.39	1486.80	2089.18	1509.98	1276.94	
Individuals	1,102		1,354		92		235		222		
Person-years	6,535		6,981		558		1,193		1,262		

To test for differences across racial/ethnic groups, we conducted ANOVA tests for continuous variables and chi-squared tests for categorical variables.

Post-hoc Tukey tests showed that compared to White individuals, White & Caribbean (*p* <.001), White & Asian (*p* <.01), White & any other mixed (*p* <.001),Pakistani (*p* <.001), Bangladeshi (*p* <.001), Caribbean (*p* <.05), Arab (*p* <.001) and any other group (*p* <.001) reported significantly higher mean mental health scores, indicating poorer mental health, while African (*p* <.001) individuals reported significantly lower mean scores, indicating better mental health; no significant differences were observed for White & African (*p* >.05), Indian (*p* >.05), Chinese (*p* >.05), any other Asian (*p* >.05), or any other Black (*p* >.05) individuals. For life satisfaction, all non-White groups—White & Caribbean (*p* <.001), White & African (*p* <.001), White & Asian (*p* <.001), White & any other mixed (*p* <.001), Indian (*p* <.001), Pakistani (*p* <.001), Bangladeshi (*p* <.001), Chinese (*p* <.001), any other Asian (*p* <.001), Caribbean (*p*< 0.001), African (*p* <.001), any other Black (*p* <.001), Arab (*p* <.001), and any other group (*p* <.001) reported significantly higher mean scores, indicating lower life satisfaction, compared to White individuals. For financial strain, White & Caribbean (*p* <.001), White & African (*p* <.001), White & Asian (*p* < 0.001), White & any other mixed (*p* <.001), Indian (*p* <.001), Pakistani (*p* <.001), Bangladeshi (*p* <.001), any other Asian (*p* <.001), Caribbean (*p* <.001), African (*p* <.001), any other Black (*p* <.001), Arab (*p* <.001), and any other group (*p* <.001) all reported significantly higher mean scores, indicating greater financial strain, while Chinese individuals did not differ significantly from White individuals (*p* >.05).

Table 1 presents the fixed-effects regression models estimating the associations between financial strain and both mental health and life satisfaction for the full sample. Model 1 indicates that financial strain is significantly associated with poorer mental health (b = 1.027, *p* <.001). Similarly, Model 2 shows that financial strain is linked to lower life satisfaction (b = 0.251, *p* <.001). These findings support Hypothesis 1, confirming that financial strain is adversely associated with both mental health and life satisfaction.

Table 2 displays fixed-effects regression models examining the associations between financial strain and both outcomes across broad racial/ethnic groups (White, Mixed, Asian, Black, and Other). In the top panel, Models 1 through 5 show that financial strain is consistently associated with poorer mental health across all groups: White (b = 1.016, *p* <.001), Mixed (b = 1.123, *p* <.001), Asian (b = 1.058, *p* <.001), Black (b = 1.096, *p* <.001), and Other racial/ethnic adults (b = 1.133, *p* <.001). The interaction analysis in Model 6 reveals that the adverse association between financial strain and mental health is significantly stronger for Black adults compared to White adults (b = 0.108, *p* <.05).

In the bottom panel of Table 2, Models 1 through 5 demonstrate that financial strain is consistently linked to lower life satisfaction across all racial/ethnic groups: White (b = 0.251, *p* <.001), Mixed (b = 0.278, *p* <.001), Asian (b = 0.238, *p* <.001), Black (b = 0.265, *p* <.001), and Other racial/ethnic adults (b = 0.214, *p* <.001). However, the interaction analysis in Model 6 shows little evidence that race/ethnicity moderates the association between financial strain and life satisfaction.

Table 3 presents additional fixed-effects regression models examining the associations between financial strain and both outcomes across more detailed racial/ethnic subgroups. In the top panel, Models 1 through 11 indicate that financial strain is consistently associated with poorer mental health across all subgroups: White and Caribbean (b = 1.351, *p* <.001), White and African (b = 1.141, *p* <.001), White and Asian (b = 0.752, *p* <.001), Indian (b = 1.108, *p* <.001), Pakistani (b = 1.120, *p* <.001), Bangladeshi (b = 1.087, *p* <.001), Chinese (b = 0.597, *p* <.001), Caribbean (b = 1.065, *p* <.001), African (b = 1.129, *p* <.001), and Arab (b = 1.342, *p* <.001) adults. The interaction analysis in Model 12 reveals nuanced results. Specifically, the adverse association between financial strain and mental health is statistically stronger for White and Caribbean (b = 0.321, *p* <.01), Indian (b = 0.110, *p* <.05), Pakistani (b = 0.105, *p* <.10), African (b = 0.140, *p* <.05), and Arab (b = 0.382, *p* <.05) adults compared to White adults. Conversely, the association is significantly weaker for White and Asian (b = − 0.314, *p* <.05) and Chinese (b = − 0.433, *p* <.05) adults compared to White adults.

In the bottom panel of Table 3, Models 1 through 11 demonstrate that financial strain is consistently linked to lower life satisfaction across all racial/ethnic minority subgroups: White and Caribbean (b = 0.278, *p* <.001), White and African (b = 0.232, *p* <.001), White and Asian (b = 0.276, *p* <.001), Indian (b = 0.254, *p* <.001), Pakistani (b = 0.219, *p* <.001), Bangladeshi (b = 0.264, *p* <.001), Chinese (b = 0.278, *p* <.001), Caribbean (b = 0.260, *p* <.001), African (b = 0.262, *p* <.001), and Arab (b = 0.200, *p* <.001) adults. However, the interaction analysis in Model 12 shows little evidence that racial/ethnic subgroup moderates the association between financial strain and life satisfaction. These findings partially support Hypothesis 2, suggesting that the adverse association between financial strain and well-being is stronger for racial/ethnic minority adults compared to White adults. Due to the number of models, the inclusion of interaction terms, and the large number of racial/ethnic subgroups, we present only the key coefficients in the main tables for readability and ease of interpretation. Full model results (including all covariates) are provided in Supplementary Appendices 1–3 for interested readers.

## Discussion and Conclusion

This study examined how within-individual changes in financial strain are associated with changes in mental health and life satisfaction, and whether these associations differ across racial/ethnic groups. Drawing on 13 waves of nationally representative panel data from the UK and employing fixed-effects regression models, we addressed several key gaps in the existing literature. These include the predominance of cross-sectional designs (Upenieks & Ellison, 2022; Walsh, 2024), the use of population-averaged models that do not account for unobserved heterogeneity (Choi et al., 2023), and the limited number of studies examining both emotional (mental health) and evaluative (life satisfaction) dimensions of well-being together. We also address the limited attention to racial/ethnic variation in the consequences of financial strain for well-being, particularly in non-U.S. contexts. Guided by the stress process model (Pearlin & Bierman, 2013) and the minority stress perspective (Meyer, 2003), our study offers a more rigorous and contextually grounded analysis of how perceived financial stress shapes well-being over time and across diverse populations.

The key findings from this study offer several important insights. First, financial strain is consistently associated with poorer mental health and lower life satisfaction. These results reinforce theoretical expectations from the stress process model (Pearlin & Bierman, 2013), which emphasizes the harmful effects of chronic stressors like financial strain on well-being. They also build on and strengthen a substantial body of cross-sectional research linking financial strain to adverse mental health outcomes (Acevedo et al., 2014; Upenieks & Ellison, 2022) and diminished life satisfaction (Jung, 2018; Walsh, 2024). Furthermore, stratified analyses show that the adverse association between financial strain and both outcomes holds for several racial/ethnic groups, including Black Caribbean and African adults—echoing findings from U.S.-based studies (Hughes et al., 2014)—as well as among adults of Mixed (e.g., White and Caribbean, White and African, White and Asian), Asian (e.g., Indian, Pakistani, Bangladeshi, Chinese), and Other backgrounds (e.g., Arab). To our knowledge, this is the first UK-based longitudinal study to document the relationship between financial strain and both mental health and life satisfaction across such a diverse set of racial/ethnic groups. These findings underscore the pervasive association of financial strain across multiple populations and point to the importance of future research incorporating additional indicators of well-being to capture the broader consequences of perceived financial stress over time.

Second, the study finds that race/ethnicity moderates the relationship between financial strain and mental health, with Black adults experiencing a more pronounced adverse association than White adults. This pattern is consistent with the stress process model (Pearlin & Bierman, 2013), which emphasizes that racial/ethnic minorities are often disproportionately exposed to chronic stressors—such as financial strain—due to systemic socioeconomic disadvantages. These structural inequalities also limit access to psychosocial resources that could otherwise buffer the negative effects of stress. The results also align with the minority stress perspective (De Leon et al., 2024; Nie, 2024), which highlights how minority-specific stressors—including experiences of racial discrimination—can compound general stress exposure and amplify psychological distress among Black adults.

By contrast, the association between financial strain and mental health does not significantly differ for Mixed, Asian, and Other racial/ethnic groups compared to White adults. Various cultural and social mechanisms may serve a protective role within these groups (e.g., Dulai & Jaspal, 2024). For instance, collectivist norms common in some Asian communities emphasize family obligation and mutual support, which may enhance resilience in the face of economic adversity (Burholt et al., 2018). Among ethnic minority groups in Scotland, perceived social support and strong minority identity have been linked to lower stress and better mental health (Heim et al., 2011). Mixed-race individuals may benefit from bicultural competence, allowing them to draw on diverse cultural frameworks and coping strategies to navigate financial strain (Caballero et al., 2008). While these mechanisms were not directly tested in this study, they may help explain why the strength of the financial strain—mental health association did not differ significantly for these groups relative to White adults.

Third, disaggregated analyses by racial/ethnic subgroup reveal important variation in the mental health consequences of financial strain. Among Black adults, the adverse association between financial strain and mental health is stronger for African adults than for White adults, whereas the association is not statistically significant for Caribbean adults. For African adults, elevated rates of economic insecurity—including higher unemployment and persistent poverty—may magnify the psychological toll of financial strain. For instance, while only 12% of White individuals in the UK live in Households with persistent low income, this figure rises to 27% among Black individuals (DWP, 2023). Structural barriers such as labor market discrimination and challenges related to the recognition of foreign credentials may further compound these disadvantages for African migrants (Zwysen et al., 2021). In contrast, Caribbean adults may benefit from more robust community-based support systems. Research suggests that Caribbean communities in the UK report greater openness toward discussing mental health, more positive experiences with services, and stronger engagement with community organizations (Rabiee-Khan & Smith, 2014). Such support may buffer the psychological consequences of financial strain, helping to explain the insignificant association for Caribbean adults.

Among Asian subgroups, we find important contrasts as well. Indian adults exhibit a significantly stronger association between financial strain and mental health than White adults, whereas this association is significantly weaker for Chinese adults. These patterns may reflect both structural inequalities and cultural expectations. Indian adults in the UK are often subject to wage gaps, insecure employment, and family-based economic obligations—such as remittances and financial support for extended kin (Batnitzky et al., 2012; Thandi, 2019). These stressors may compound feelings of financial strain, especially in the context of intergenerational expectations and norms emphasizing familial responsibility (Kaur, 2019; Verma et al., 2024). In contrast, Chinese adults may experience weaker mental health consequences of financial strain due to cultural values that emphasize endurance, restraint, and resilience (Chan, 2009; Ho et al., 2021). These norms may serve as internal coping resources that help mitigate the emotional toll of financial strain.

The results also indicate a stronger association between financial strain and poorer mental health for Pakistani and Arab adults compared to White adults. Among Pakistani adults, socioeconomic disadvantage, unstable employment, and limited access to culturally responsive mental health services likely exacerbate the psychological consequences of financial strain (Burholt et al., 2018; Clark & Shankley, 2020). Additionally, cultural norms emphasizing financial responsibility to extended families may heighten economic pressure. For Arab adults, experiences of discrimination, Islamophobia, and structural barriers to integration (Hackett et al., 2020), combined with mental health stigma within some communities (Hamid & Furnham, 2013), may increase psychological vulnerability in the face of financial stress. These intersectional stressors are often overlooked in mainstream health frameworks but appear to intensify the effects of economic hardship (Chow, 2025).

Together, these findings underscore the importance of disaggregating broad racial/ethnic categories to reveal subgroup-specific dynamics. Aggregated categories such as “Asian” or “Black” obscure important heterogeneity in socioeconomic context, migration history, cultural values, and access to support. Disaggregation enables a clearer understanding of which groups are most vulnerable and why—insights that are essential for tailoring culturally relevant policy and practice interventions. Future research should continue to investigate how cultural, structural, and psychosocial factors interact to shape the mental health consequences of financial strain within and across diverse racial/ethnic communities.

Fourth, the association between financial strain and life satisfaction does not differ significantly across racial/ethnic groups. This suggests that financial strain may be similarly related to life satisfaction across groups, even though its association with mental health varies. One possible explanation lies in the nature of life satisfaction itself, which, as a broader and more stable evaluation of one’s overall circumstances—rather than a reflection of immediate emotional states—is shaped by a wide range of life domains, including family relationships, job satisfaction, and long-term goals (Pavot & Diener, 2008). These broader domains may help buffer financial stress in ways that are less susceptible to minority-specific stressors such as discrimination or stigma. Moreover, financial strain may be similarly linked to life satisfaction across groups because it threatens basic needs and aspirations that are universally important. Regardless of race or ethnicity, individuals may report lower life satisfaction when financial stress interfere with affording essentials, achieving personal goals, or maintaining a desired standard of living, and these shared constraints may explain the relatively uniform association between financial strain and life satisfaction across racial/ethnic groups.

## Limitations

Several limitations warrant acknowledgment. First, although this study uses longitudinal data, the potential for reverse causality cannot be ruled out. Financial strain and the outcome variables (mental health and life satisfaction) were measured within the same survey waves, meaning financial strain was not assessed temporally prior to the outcomes. As a result, the directionality of associations cannot be definitively established. However, our use of fixed-effects models strengthens the robustness of the findings by controlling for all time-invariant individual characteristics (e.g., personality traits) and focusing on within-individual changes across time. This analytical approach provides stronger evidence than cross-sectional models, though it does not resolve concerns around simultaneity. Indeed, the associations observed may be bidirectional—for instance, financial strain may contribute to poorer mental health, but poor mental health may also heighten financial strain by impairing individuals’ ability to work or manage resources. Future research should consider longitudinal designs with lagged measures or cross-lagged panel models to better disentangle these reciprocal pathways.

Second, although widely used in population research, the single-item measures of financial strain may not fully capture the complexity of this construct. Future studies would benefit from using multiple-item scales (Chai et al., 2021; Choi et al., 2021; Jung, 2018) to better capture the multidimensional nature of financial strain and improve measurement prevision. Third, immigration status may present an important time-varying confounder in the association between financial strain and well-being. However, this information was not continuously collected in the UKHLS, limiting its utility in the current study. Future research should incorporate this factor where possible, as immigration-related barriers—such as legal status or acculturative stress—may compound financial strain and influence well-being in distinct ways.

## Contributions

Despite these limitations, this study offers several contributions. First, it is among the few to examine the longitudinal association between financial strain and both mental health and life satisfaction using fixed-effects models and a large, nationally representative dataset. This approach strengthens the findings by addressing unobserved heterogeneity that may bias cross-sectional associations. Second, the study explicitly tests for racial/ethnic moderation and disaggregates broad racial/ethnic categories into more specific subgroups, revealing meaningful variations that would be obscured in aggregate comparisons. These findings advance understanding of how subgroup-specific socioeconomic conditions and cultural dynamics may shape the relationship between financial strain and well-being. Third, by simultaneously analyzing mental health and life satisfaction, the study offers a more holistic assessment of well-being outcomes. This dual focus enables a nuanced interpretation of how financial strain relates to both emotional functioning and broader life evaluations—dimensions that may not always align.

Together, these contributions suggest that financial strain is an important determinant of well-being across racial/ethnic groups and emphasize the need for policy responses that are both economically and culturally informed. Future research should build on this work by incorporating psychosocial mediators, examining life course variations, and developing interventions that are responsive to the structural and cultural contexts in which financial stress is experienced.

## Supplementary Information

Below is the link to the electronic supplementary material.

Supplemetary File 1 (DOCX 28.0KB)
