# Supplementary material for: Racial/ethnic Variations in the Association Between Financial Strain and Well-Being: Evidence from the United Kingdom Household Longitudinal Survey
Source: Appl Res Qual Life. 2025 Oct 24;20(5):2049–77. doi: 10.1007/s11482-025-10502-5 (PMC12678589; doi:10.1007/s11482-025-10502-5)
Supplement: Supplementary file 1 — Supplemetary File 1 (DOCX 28.0KB) [file 11482_2025_10502_MOESM1_ESM.docx]

| Appendix 1. Fixed-effects regression models predicting | | |
| --- | --- | --- |
| mental health and life satisfaction. | | |
|  | Model 1 | Model 2 |
|  | Mental | Life |
|  | health | satisfaction |
|  | b | b |
| Financial strain | 1.027*** | .251*** |
|  | (.010) | (.003) |
| Marital status |  |  |
| Married | .009 | -.034*** |
|  | (.035) | (.010) |
| Unmarried (ref.) |  |  |
| Number of children | -.038 | -.011 |
|  | (.022) | (.006) |
| Education |  |  |
| High (ref.) |  |  |
| Low | -.607*** | -.145*** |
|  | (.059) | (.017) |
| Employment status |  |  |
| Employed | -.383*** | .031*** |
|  | (.026) | (.007) |
| Other (ref.) |  |  |
| Personal income (logged) | -.012* | -.000 |
|  | (.006) | (.002) |
| Individuals | 58,029 | 58,029 |
| Person-years | 417,766 | 417,766 |
| Note: All models include a full set of control variables: | | |
| marital status, number of children, education, | | |
| employment status, personal income (logged), and | | |
| survey-year. Education was recoded into a binary | | |
| variable, distinguishing individuals with a bachelor’s | | |
| degree or higher from those with lower educational | | |
| attainment, to ensure sufficient cell sizes when | | |
| examining racial/ethnic differences. b = coefficient. | | |
| Standard errors are in parentheses. ****p* < .001; **p* < .05. | | |

| Appendix 2. Fixed-effects regression models predicting mental health and life satisfaction. | | | | | | |  |
| --- | --- | --- | --- | --- | --- | --- | --- |
|  | Model 1 | Model 2 | Model 3 | Model 4 | Model 5 | Model 6 |  |
|  | Top panel: Mental health | | | | | |  |
|  | White | Mixed | Asian | Black | Other | Full |  |
|  | b | b | b | b | b | b |  |
| Financial strain | 1.016*** | 1.123*** | 1.058*** | 1.096*** | 1.133*** | 1.014*** |  |
|  | (.011) | (.082) | (.036) | (.053) | (.133) | (.011) |  |
| Financial strain X race/ethnicity |  |  |  |  |  |  |  |
| Financial strain X Mixed |  |  |  |  |  | .080 |  |
|  |  |  |  |  |  | (.072) |  |
| Financial strain X Asian |  |  |  |  |  | .056 |  |
|  |  |  |  |  |  | (.034) |  |
| Financial strain X Black |  |  |  |  |  | .108* |  |
|  |  |  |  |  |  | (.049) |  |
| Financial strain X Other |  |  |  |  |  | .167 |  |
|  |  |  |  |  |  | (.117) |  |
| Marital status |  |  |  |  |  |  |  |
| Married | .017 | .022 | -.195 | .163 | .237 | .008 |  |
|  | (.036) | (.329) | (.148) | (.200) | (.547) | (.035) |  |
| Unmarried (ref.) |  |  |  |  |  |  |  |
| Number of children | -.066** | .764*** | -.013 | .109 | -.095 | -.038 |  |
|  | (.024) | (.191) | (.069) | (.108) | (.283) | (.022) |  |
| Education |  |  |  |  |  |  |  |
| High (ref.) |  |  |  |  |  |  |  |
| Low | -.559*** | -.713 | -1.224*** | -.039 | -.733 | -.607*** |  |
|  | (.065) | (.370) | (.191) | (.278) | (.812) | (.059) |  |
| Employment status |  |  |  |  |  |  |  |
| Employed | -.331*** | -.559** | -.705*** | -.623*** | -1.143** | -.383*** |  |
|  | (.027) | (.205) | (.099) | (.138) | (.371) | (.026) |  |
| Other (ref.) |  |  |  |  |  |  |  |
| Personal income (logged) | -.013* | .060 | .008 | -.048 | -.046 | -.012* |  |
|  | (.007) | (.043) | (.016) | (.028) | (.059) | (.006) |  |
|  | Bottom panel: Life satisfaction | | | | | |  |
|  | White | Mixed | Asian | Black | Other | Full |  |
|  | b | b | b | b | b | b |  |
| Financial strain | .251*** | .278*** | .238*** | .265*** | .214*** | .250*** |  |
|  | (.003) | (.021) | (.010) | (.015) | (.036) | (.003) |  |
| Financial strain X race/ethnicity |  |  |  |  |  |  |  |
| Financial strain X Mixed |  |  |  |  |  | .025 |  |
|  |  |  |  |  |  | (.021) |  |
| Financial strain X Asian |  |  |  |  |  | -.005 |  |
|  |  |  |  |  |  | (.010) |  |
| Financial strain X Black |  |  |  |  |  | .025 |  |
|  |  |  |  |  |  | (.014) |  |
| Financial strain X Other |  |  |  |  |  | -.020 |  |
|  |  |  |  |  |  | (.033) |  |
| Marital status |  |  |  |  |  |  |  |
| Married | -.027* | -.101 | -.126** | .018 | -.202 | -.034*** |  |
|  | (.010) | (083) | (.041) | (.057) | (.149) | (.010) |  |
| Unmarried (ref.) |  |  |  |  |  |  |  |
| Number of children | -.020** | .113* | .006 | .052 | -.067 | -.011 |  |
|  | (.007) | (.048) | (.019) | (.031) | (.077) | (.006) |  |
| Education |  |  |  |  |  |  |  |
| High (ref.) |  |  |  |  |  |  |  |
| Low | -.140*** | -.043 | -.215*** | -.190* | -.180 | -.145*** |  |
|  | (.019) | (.094) | (.053) | (.080) | (.221) | (.017) |  |
| Employment status |  |  |  |  |  |  |  |
| Employed | .038*** | -.026 | -.030 | .095* | -.150 | .031*** |  |
|  | (.008) | (.052) | (.027) | (.040) | (.101) | (.007) |  |
| Other (ref.) |  |  |  |  |  |  |  |
| Personal income (logged) | -.001 | .017 | .005 | -.004 | -.014 | .000 |  |
|  | (.002) | (.011) | (.004) | (.008) | (.016) | (.002) |  |
| Individuals | 48,076 | 1,084 | 5,864 | 2,548 | 457 | 58,029 |  |
| Person-years | 361,161 | 6,737 | 33,339 | 14,074 | 2,455 | 417,766 |  |
| Note: All models include a full set of control variables: marital status, number of children, education, employment | | | | | | | |
| status, personal income (logged), and survey-year. Education was recoded into a binary variable distinguishing | | | | | | | |
| individuals with a bachelor’s degree or higher from those with lower educational attainment, to ensure sufficient | | | | | | | |
| cell sizes when examining racial/ethnic differences. b = coefficient. Standard errors are in parentheses. | | | | | | | |
| ****p* < .001; ***p* < .10; **p* < .05. | | | | | | | |

| Appendix 3. Fixed-effects regression models predicting mental health and life satisfaction | | | | | | | | | | | | | |
| --- | --- | --- | --- | --- | --- | --- | --- | --- | --- | --- | --- | --- | --- |
|  | Model 1 | Model 2 | Model 3 | Model 4 | Model 5 | Model 6 | Model 7 | Model 8 | Model 9 | Model 10 | Model 11 | Model 12 |  |
|  | Top panel: Mental health | | | | | | | | | | | | |
|  | White | White & | White & | White & | Indian | Pakistani | Bangladeshi | Chinese | Caribbean | African | Arab | Full |  |
|  |  | Caribbean | African | Asian |  |  |  |  |  |  |  |  |  |
|  | b | b | b | b | b | b | b | b | b | b | b | b |  |
| Financial strain | 1.016*** | 1.351*** | 1.141*** | .752*** | 1.108*** | 1.120*** | 1.087*** | .597*** | 1.065*** | 1.129*** | 1.342*** | 1.014*** |  |
|  | (.011) | (.132) | (.213) | (.162) | (.058) | (.067) | (.090) | (.169) | (.079) | (.073) | (.189) | (.011) |  |
| Financial strain X race/ethnicity | | | |  |  |  |  |  |  |  |  |  |  |
|  |  |  |  |  |  |  |  |  |  |  |  |  |  |
| Financial strain X White & Caribbean | | |  |  |  |  |  |  |  |  |  | .321** |  |
|  |  |  |  |  |  |  |  |  |  |  |  | (.112) |  |
| Financial strain X White & African | | |  |  |  |  |  |  |  |  |  | .097 |  |
|  |  |  |  |  |  |  |  |  |  |  |  | (.197) |  |
| Financial strain X White & Asian | |  |  |  |  |  |  |  |  |  |  | -.314* |  |
|  |  |  |  |  |  |  |  |  |  |  |  | (.148) |  |
| Financial strain X Indian | | |  |  |  |  |  |  |  |  |  | .110* |  |
|  |  |  |  |  |  |  |  |  |  |  |  | (.054) |  |
| Financial strain X Pakistani | |  |  |  |  |  |  |  |  |  |  | .105+ |  |
|  |  |  |  |  |  |  |  |  |  |  |  | (.056) |  |
| Financial strain X Bangladeshi | | |  |  |  |  |  |  |  |  |  | .100 |  |
|  |  |  |  |  |  |  |  |  |  |  |  | (.077) |  |
| Financial strain X Chinese | | |  |  |  |  |  |  |  |  |  | -.433* |  |
|  |  |  |  |  |  |  |  |  |  |  |  | (.175) |  |
| Financial strain X Caribbean | |  |  |  |  |  |  |  |  |  |  | .096 |  |
|  |  |  |  |  |  |  |  |  |  |  |  | (.073) |  |
| Financial strain X African | | |  |  |  |  |  |  |  |  |  | .140* |  |
|  |  |  |  |  |  |  |  |  |  |  |  | (.068) |  |
| Financial strain X Arab | | |  |  |  |  |  |  |  |  |  | .382* |  |
|  |  |  |  |  |  |  |  |  |  |  |  | (.167) |  |
| Marital status |  |  |  |  |  |  |  |  |  |  |  |  |  |
| Married | .017 | .209 | .204 | -.232 | -.203 | -.530+ | .802* | -.088 | .373 | .022 | -.207 | .009 |  |
|  | (.036) | (.535) | (.808) | (.579) | (.250) | (.273) | (358) | (.513) | (.342) | (.254) | (.800) | (.035) |  |
| Unmarried (ref.) |  |  |  |  |  |  |  |  |  |  |  |  |  |
| Number of children | -.066** | .748* | .995* | .615 | .004 | .034 | -.097 | -.500 | .197 | .046 | -.073 | -.038+ |  |
|  | (.024) | (.305) | (.434) | (.389) | (.121) | (.117) | (.172) | (.313) | (.185) | (.137) | (.436) | (.022) |  |
| Education |  |  |  |  |  |  |  |  |  |  |  |  |  |
| High (ref.) |  |  |  |  |  |  |  |  |  |  |  |  |  |
| Low | -.599*** | -1.430* | -2.033* | 1.604* | -.665* | -1.396*** | -1.570*** | -.139 | -.254 | -.017 | -1.296 | -.608*** |  |
|  | (.065) | (.595) | (.961) | (.759) | (.336) | (.360) | (.419) | (.814) | (.477) | (.360) | (1.066) | (.059) |  |
| Employment status |  |  |  |  |  |  |  |  |  |  |  |  |  |
| Employed | -.331*** | -1.148*** | -.659 | -.162 | -1.109*** | -.531** | -.570* | .373 | -.899*** | -.414* | -1.264* | -.383*** |  |
|  | (.027) | (.340) | (.530) | (.388) | (.158) | (.196) | (.244) | (.432) | (.206) | (.194) | (.534) | (.026) |  |
| Other (ref.) |  |  |  |  |  |  |  |  |  |  |  |  |  |
| Personal income (logged) | -.013* | .229** | .077 | -.211* | .052* | .009 | -.059 | -.031 | -.058 | -.048 | -.049 | -.012* |  |
|  | (.007) | (.076) | (.115) | (.082) | (.025) | (.029) | (.038) | (.076) | (.046) | (.036) | (.078) | (.006) |  |
|  | Bottom panel: Life satisfaction | | | | | | | | | | | | |
|  | White | White & | White & | White & | Indian | Pakistani | Bangladeshi | Chinese | Caribbean | African | Arab | Full |  |
|  |  | Caribbean | African | Asian |  |  |  |  |  |  |  |  |  |
|  | b | b | b | b | b | b | b | b | b | b | b | b |  |
| Financial strain | .251*** | .278*** | .232*** | .276*** | .254*** | .219*** | .264*** | .278*** | .260*** | .262*** | .200*** | .250*** |  |
|  | (.003) | (.033) | (.061) | (.041) | (.016) | (.018) | (.025) | (.046) | (.023) | (.021) | (.054) | (.003) |  |
| Financial strain X race/ethnicity | |  |  |  |  |  |  |  |  |  |  |  |  |
|  |  |  |  |  |  |  |  |  |  |  |  |  |  |
| Financial strain X White & Caribbean | | |  |  |  |  |  |  |  |  |  | .030 |  |
|  |  |  |  |  |  |  |  |  |  |  |  | (.032) |  |
| Financial strain X White & African | | |  |  |  |  |  |  |  |  |  | -.007 |  |
|  |  |  |  |  |  |  |  |  |  |  |  | (.056) |  |
| Financial strain X White & Asian | |  |  |  |  |  |  |  |  |  |  | .016 |  |
|  |  |  |  |  |  |  |  |  |  |  |  | (.042) |  |
| Financial strain X Indian | |  |  |  |  |  |  |  |  |  |  | .013 |  |
|  |  |  |  |  |  |  |  |  |  |  |  | (.015) |  |
| Financial strain X Pakistani | |  |  |  |  |  |  |  |  |  |  | -.023 |  |
|  |  |  |  |  |  |  |  |  |  |  |  | (.016) |  |
| Financial strain X Bangladeshi | | |  |  |  |  |  |  |  |  |  | .018 |  |
|  |  |  |  |  |  |  |  |  |  |  |  | (.022) |  |
| Financial strain X Chinese | | |  |  |  |  |  |  |  |  |  | .022 |  |
|  |  |  |  |  |  |  |  |  |  |  |  | (.050) |  |
| Financial strain X Caribbean | |  |  |  |  |  |  |  |  |  |  | .026 |  |
|  |  |  |  |  |  |  |  |  |  |  |  | (.021) |  |
| Financial strain X African | | |  |  |  |  |  |  |  |  |  | .021 |  |
|  |  |  |  |  |  |  |  |  |  |  |  | (.019) |  |
| Financial strain X Arab | | |  |  |  |  |  |  |  |  |  | -.049 |  |
|  |  |  |  |  |  |  |  |  |  |  |  | (.047) |  |
| Marital status |  |  |  |  |  |  |  |  |  |  |  |  |  |
| Married | -.027* | -.041 | -.290 | -.077 | -.195** | -.035 | -.100 | -.199 | -.108 | .070 | -.323 | -.034*** |  |
|  | (.010) | (.136) | (.230) | (.147) | (.068) | (.075) | (.099) | (.139) | (.098) | (.073) | (.228) | (.010) |  |
| Unmarried (ref.) |  |  |  |  |  |  |  |  |  |  |  |  |  |
| Number of children | -.020** | .116 | .178 | .077 | .059+ | .007 | -.061 | -.020 | .060 | .042 | .127 | -.012+ |  |
|  | (.007) | (.078) | (.123) | (.099) | (.033) | (.032) | (.048) | (.085) | (.053) | (.039) | (.124) | (.006) |  |
| Education |  |  |  |  |  |  |  |  |  |  |  |  |  |
| High (ref.) |  |  |  |  |  |  |  |  |  |  |  |  |  |
| Low | -.140*** | .036 | -.038 | .105 | -.281** | -.241* | -.128 | -.072 | -.243+ | -.178+ | -.284 | -.145*** |  |
|  | (.019) | (.151) | (.273) | (.192) | (.092) | (.098) | (.116) | (.220) | (.137) | (.103) | (.304) | (.017) |  |
| Employment status |  |  |  |  |  |  |  |  |  |  |  |  |  |
| Employed | .038*** | -.169* | .054 | .143 | -.101* | -.017 | -.001 | .214+ | .011 | .148** | -.143 | .031*** |  |
|  | (.008) | (.086) | (.151) | (.098) | (.043) | (.054) | (.068) | (.117) | (.059) | (.056) | (.152) | (.007) |  |
| Other (ref.) |  |  |  |  |  |  |  |  |  |  |  |  |  |
| Personal income (logged) | -.001 | .058** | -.050 | -.016 | .010 | .003 | -.012 | .000 | .005 | -.010 | -.003 | -.000 |  |
|  | (.002) | (.019) | (.033) | (.021) | (.007) | (.008) | (.010) | (.021) | (.013) | (.010) | (.022) | (.002) |  |
| Individuals | 48,076 | 430 | 159 | 255 | 2,155 | 1,832 | 1,024 | 238 | 1,102 | 1,354 | 235 | 58,029 |  |
| Person-years | 361,161 | 2,694 | 911 | 1,685 | 13,008 | 9,851 | 5,211 | 1,643 | 6,535 | 6,981 | 1,193 | 417,766 |  |
| Note: All models include a full set of control variables: marital status, number of children, education, employment status, personal income (logged), and survey-year. | | | | | | | | | | | | | |
| Education was recoded into a binary variable, distinguishing individuals with a bachelor’s degree or higher from those with lower educational attainment, to ensure sufficient | | | | | | | | | | | | | |
| cell sizes when examining racial/ethnic differences. b = coefficient. Standard errors are in parentheses. ****p* < .001; ***p* < .01; **p* < .05; +*p* < .10. | | | | | | | | | | | | | |
| The following categories were excluded due to limited table space and challenges in interpreting these categories: any other mixed, any other Asian, any other Black | | | | | | | | | | | | | |
| , and any other race/ethnicity. Due to smaller subgroup sizes in Table 5, a significance threshold of .10 was used, as smaller samples reduce statistical power | | | | | | | | | | | | | |
| to detect effects at the conventional 0.05 level. | | | | | | | | | | | | | |
